# Dry needling in active or latent trigger point in patients with neck pain: a randomized clinical trial

**DOI:** 10.1038/s41598-022-07063-0

**Published:** 2022-02-24

**Authors:** Luis Martín-Sacristán, Cesar Calvo-Lobo, Daniel Pecos-Martín, Josué Fernández-Carnero, José Luis Alonso-Pérez

**Affiliations:** 1 Department of Physical Therapy, Alcalá University, Alcalá de Henares, Madrid Spain; 2 Faculty of Nursing, Physiotherapy and Podiatry, Complutense University of Madrid, Madrid, Spain; 3 Physiotherapy and Pain Group, Department of Physical Therapy, Occupational Therapy, Rehabilitation and Physical Medicine, Rey Juan Carlos University, Móstoles, Madrid, Spain; 4 La Paz Hospital Institute for Health Research, IdiPAZ, Madrid, Spain; 5 Grupo Multidisciplinar de Investigación y Tratamiento del Dolor, Grupo de Excelencia Investigadora, URJC-Banco de Santander, Madrid, Spain; 6 Universidad Europea de Madrid, Faculty of Sport Sciences, Villaviciosa de Odón, 28670 Madrid, Spain; 7 Universidad Europea de Canarias, Faculty of Health Sciences, La Orotava, 38300 Tenerife, Canary Islands Spain; 8 Musculoskeletal Pain and Motor Control Research Group, Faculty of Sport Sciences, Universidad Europea de Madrid, Madrid, Spain; 9 Musculoskeletal Pain and Motor Control Research Group, Faculty of Health Sciences, Universidad Europea de Canarias, Santa Cruz de Tenerife, Spain

**Keywords:** Musculoskeletal system, Diseases, Health care, Medical research, Rheumatology

## Abstract

The purpose was to determine the efficacy of deep dry needling (DDN) applied on an active myofascial trigger point (MTrP) versus a latent-MTrP versus a non-MTrP location, on pain reduction and cervical disability, in patients with chronic neck pain. A randomized, double-blind clinical trial design was used. A sample of 65 patients was divided into non-MTrP-DDN, active-MTrP-DDN and latent-MTrP-DDN groups. The visual analog scale (VAS), reproduction of the patient’s pain, number of local twitch responses, pressure pain threshold (PPT) and Neck Disability Index (NDI) were assessed before, during and after the intervention and up to 1 month post-intervention. The active-MTrP-DDN-group reduced pain intensity more than non-MTrP-DDN-group after a week and a month (P < 0.01), as well as showing the greatest improvement in tibialis muscle PPT. The treatment of both Active and Latent MTrPs was associated with the reproduction of the patient’s pain. The application of DDN on an active-MTrP in the upper trapezius muscle shows greater improvements in pain intensity after 1 week and 1 month post-intervention, compared to DDN applied in latent-MTrPs or outside of MTrPs in patients with neck pain.

## Introduction

Neck pain is the fourth leading cause of disability, with an annual prevalence rate exceeding 30%. In the world, around 70% of the population will have neck pain throughout their lives, while the prevalence in Spain is 19.5% and is higher in women than men^1,2^. Although most cases of acute neck pain seem to resolve with or without treatment, but up to 50% of people who have it may continue feeling pain or have frequent pain episodes^2–4^. In addition, there are several important factors that are related to the appearance of neck pain, especially those related to work, stress and depression^1^. In relation to work, neck pain appears more frequently in those jobs which involving prolonged static postures and repetitive movements of the upper limbs^5^.

It has also been proposed that myofascial trigger points (MTrPs) could be the responsible for the development of pain in patients with mechanical neck pain; therefore, some studies include MTrP therapy for the management of these patients^6^. The prevalence of MTrPs in patients with myofascial pain was 93.75% for the upper trapezius muscle, being the most prevalent muscle for MTrPs presence. Within the same, the prevalence of active MTrP was higher on the right side with 82.1% while on the left side it was 79%^4^. The reason why the upper trapezius is the muscle that most frequently presents MTrPs of the whole body is due to its permanent activity and micro-trauma^7^.

Myofascial trigger point is defined as a hypersensitive spot in a taut band of a skeletal muscle and referred pain while myofascial pain syndrome (MPS) can be diagnosed by the presence of one or more MTrPs^8,9^. MTrPs are clinically classified as active or latent. Active MTrPs are those that cause spontaneous pain or pain during movement, stretching or compression. Latent MTrPs are usually asymptomatic, but they reproduce pain or discomfort when they are compressed^5,9^. Other typical symptoms associated with MTrPs in addition to local and referred pain is the appearance of muscle weakness and a restricted range of motion. In the case of active and latent MTrPs, both elicit a local twitch response (LTR) and pain that reproduces the patient's symptoms^9,10^. The combination of these symptoms could have a large impact on the quality of life, mood, and health status^5^.

Among the treatment of myofascial pain, this is based on the inactivation of MTrPs through manual techniques or applying the invasive technique known as dry needling (DN)^11^. Within the types of DN: the most commonly used to the MTrPs treatment is deep DN (DDN), which involves inserting a thin filiform needle directly into the muscle and through the MTrP^12,13^. Traditionally, the effectiveness of dry needling has been related to the provocation of the local twitch response (LTR). The LTR is characterized by a visible contraction of part of the taut band in the involved muscle upon mechanical stimulation with needling to a sensitive site in the MTrP area. Although there are different points of view about this, some authors suggest that MTrP DN is most effective if a LTR is elicited during the procedure^14^. To carry out the intervention, the needle is moved up and down into the muscle tissue to elicit LTRs^15^. Although there is a lack of knowledge about the mechanisms of dry needling, some studies support that LTR is crucial to achieve the elimination of pro-nociceptive and pro-inflammatory substances. However, in clinical practice, even when LTR is induced, there is not always a positive effect^16^. Some studies have shown the benefit of applying DN to an active MTrP in the trapezius muscle producing greater improvements in pain measured with visual analogue scale (VAS), pressure pain threshold (PPT) and levels of disability produced by neck pain^17^.

In relation to the DN mechanisms of action, some factors were involved in the activation of the central nervous system: complex endogenous pain modulating mechanisms and the activation of the autonomic nervous system to produce electromyographic changes in the MTrPs. This supposes a possible action on the etiopathogenesis of MTrPs, which could justify motor effects, beyond the purely sensitive ones^18,19^. Additionally, there is a sympathetic interaction with myofascial tension that demonstrates the facilitating contribution of sympathetic hyperactivity to mechanical sensitization, and the possible underlying mechanisms of local and referred muscle pain^20^. Moreover, vasoconstriction, cold skin, sweating, and pilomotor response in patients with pain due to active MTrPs may be related to sympathetic hyperactivity. The vasoconstrictor activity of the sympathetic nervous system (SNS) was activated by nociceptive stimulation of latent MTrPs^21^. Finally, another study identifies the neuromuscular junction as a target of the SNS and shows that sympathetic input is crucial for the maintenance and function of the synapse^22^.

It is also important to decide the dose of DN treatment. On this matter, some studies explain that the greater the number of “in-and-outs” with the needle in the muscle, the greater the post-needling pain appears, but, in turn, the better results seem to be found when six or more LTRs are achieved^23,24^. However, another study indicates that performing DN without eliciting LTRs has superiority over the DN eliciting LTR in relation with the treatment long-term effects^7^.

Therefore, the aim of this study was to determine the efficacy of DDN applied on an active MTrP versus a latent MTrP versus a non-MTrP location, on pain reduction, mechanical hyperalgesia and cervical disability, in patients with chronic neck pain attributable to myofascial pain syndrome. The second aim was to evaluate the association between number of LTR and improvement and the third aim was to evaluate the association between reproduce the patient’s pain during dry needling with the improvement of pain and function following dry needling.

## Materials and methods

### Study design

A randomized, double-blind clinical trial design was used for the study. Patients were randomly allocated to one of three different intervention groups (Non-MTrP group, Active-MTrP group, and Latent-MTrP group) performed by a computer-generated random-sequence table created before the start of the study with Epidat 4.2 software (Epidemiology Service of the General Directorate of Health Public of the Consellería de Sanidade (Xunta de Galicia) with the support of the Pan American Health Organization (PAHO-WHO) and the CES University of Colombia)^25^.

All subjects signed an informed consent, and the study was approved by the Ethics Committee for Animal Research and Experimentation at the University of Alcalá with the number identification CEID/HU/2018/41.

The study was guided by the Consolidated Standards of Reporting Trials (CONSORT) statement and included the CONSORT checklist.

This study was registered at NIH U.S. National Library of Medicine ClinicalTrials.gov under identifier (NCT number): NCT03345238. The study start date was registered on 13/03/2017 and the study completion date was registered on 10/08/2019.

### Participants

Participants were recruited from a Primary Health Care Centre in Alcalá de Henares (Madrid, Spain) and the University community from the University of Alcalá. Patients with neck pain of muscular origin were referred and screened for possible eligibility criteria.

In this research we defined neck pain as mechanical pain in the cervical muscles, which can be provoked both by maintained postures and by movements. Patients were selected if they met all of the following criteria: Non-specific neck pain, unilateral or bilateral, Neck pain ≥ 3 months of duration, Presence of active and latent MTP in the upper, left, right or bilateral trapezius muscle, in relation to the patient's neck pain^26^ and Patients were between 18 and 65 years of age^27^.

### Detection of active or latent MTrPs or non-MTrPs

The recent clinical criteria recommended identifying active and latent MTrPs^8,17,23,28^, such as taut band palpable, a hypersensitive spot at the pressure of a taut band, referred pain and the reproduction of any of the symptoms experienced by a patient and the recognition of pain will identify as active MTrP. It is considered positive when 2 of the clinical criteria are found^8^.

In order to meet the criteria to participate in the study, patients had to pass an initial physical examination performed by a single investigator to rule out the exclusion criteria: Unsurpassed fear of needles, Coagulation disorders, Specific alterations of the cervical region in the clinical history, Infiltration of corticosteroids or local anaesthetics during a year before the study, Surgical intervention of the cervical region or previous shoulder, Skin lesions in the area, as well as infection or inflammation, Taking analgesic, anti-inflammatory or anticoagulant medication the week before the study, Treatment of MTrP or DDN in the neck region in the 6 months prior to the intervention, Cognitive deficit in the medical history.

Each participant received a thorough explanation about the content and purpose of the treatment before signing an informed consent form relative to the procedures.

### Masking

Participants were randomized into each intervention group without knowing which of the three groups they belonged to. For the masking of the practitioner physiotherapist, the evaluator therapist marked on the skin of each participant the place where the physiotherapist had to perform the DN without the latter knowing which group each of the participants belonged to.

### Outcome measurement

#### Primary outcome measures

The primary outcome measure was neck pain intensity.

#### Neck pain intensity

Neck pain intensity was measured before, during, immediately after intervention and the follow up was at 1, 6, 12, 24, 48, 72 h after intervention and also 1 week and 1 month after intervention.

A visual analogue scale (VAS) was used, which is a well-established and validated self-report to measure the intensity of pain. It consists of a 100 mm line in which 0 indicates no pain and 100 indicate the worst pain imaginable by the patient. The patients should indicate where their pain is generally located within the measure described above. The VAS presented a moderate to good reliability (intraclass correlation coefficient 0.60 to 0.77) for the assessment of disability in patients with chronic musculoskeletal pain^29^.

#### Factors analysed during the intervention

The two factors that were assessed during the intervention were patient’s neck pain recognition and number of local twitches responses.

#### Patient’s neck pain recognition

The association between the DDN site and the provocation of the patient’s neck pain was assessed by asking the subjects of each group if their pain was reproduced and recognized during the intervention and the answers were collected as a dichotomous variable (yes/no).

#### Number of local twitches responses (LTRs)

The LTRs number was evaluated by the physiotherapist who performed the intervention on an “n” number of up to 12 dry needling in and outs in the upper trapezius muscle (n/12)^7,17,23,24^.

#### Secondary outcome measures

The secondary outcomes were neck disability and pressure pain threshold.

#### Neck disability

The validated Spanish version of the Neck Disability Index (NDI) was used to assess the degree of disability before, 1 week and 1 month after the intervention. The NDI is a valid tool for the measurement of pain and self-assessment of cervical disability. The NDI is composed of 10 questions related to daily functional activities. Each of the sections (intensity of neck pain, personal care, weight lifting, reading, headache, ability to concentrate, work capacity, driving, sleep and leisure activities) offers 6 possible answers that represent 6 levels progressive functional capacity, and it is scored from 0 to 5 (0 = no disability, 5 = total disability). The total score is expressed as a percentage of the maximum possible. A score between 5 and 14 represents a mild disability, whereas a score between 15 and 24 is interpreted as a moderate disability. Neck Disability Index scores > 25 reflect a severe disability and the maximum score is 50. NDI presents an acceptable reliability with an intraclass correlation coefficient (ICC) ranging from 0.50 to 0.98^30^.

#### Pressure pain threshold (PPT)

PPT was evaluated using a digital algometer. Pressure pain is defined as the minimum pressure that induces pain or discomfort with a 1 cm^2^ rubber disc. The results serve as a reference for the clinical diagnosis of abnormal sensitivity and for documentation of treatment results in the MPS. Pressure is applied at a rate of 1 kg/s. Three consecutive tests were performed on the active, latent and non MTrP of the upper trapezius muscle at 30 s intervals before and immediately after the intervention. Intra-evaluator reliability is high in the upper trapezius muscle (ICC = 0.94–0.97)^31^. PPT was also assessed in the tibialis anterior muscle in order to assess mechanosensitivity outside of the painful region, which showed an excellent test–retest reliability over tibialis anterior muscle (ICC = 0.71–0.91) in patients with chronic neck pain^32^. Furthermore, PPT expansion was calculated by the summation of the described pressure pain thresholds, including upper trapezius PPT as the painful region measurement and tibialis anterior PPT as the measurement outside of the painful region^31,32^.

#### Procedures and intervention

Participants were randomly assigned to the non-MTrP group or to the active MTrP group or to the latent MTrP group using randomization. Each participant was informed about the study and filled in the informed consent sheet as well as the data sheet and the NDI. After collecting the information from the participant, the evaluating therapist performed the physical evaluation of the patient and determined the major dry needling site according to the assigned group where the practitioner therapist performed dry needling intervention. Participants did not know which group they belonged to. Next, the practitioner therapist performed the dry needling intervention without knowing the group to which each participant was assigned, and again the evaluating therapist performed the post-intervention evaluations. One session of intervention were performed in each patient.

Patients were followed up for up to 1 month according to each measurement variable.

#### Dry needling

Patients were asked to lie in a supine position with the arms next to the body. Dry needling was performed with a 0.30 × 30 mm DN needle (APS Regular Agupunt) with a guiding tube. The evaluator therapist palpated the MTrPs in experimental groups and non-MTrPs group, for this group the non-MTrP site, without any type of symptomatology, a 2 cm of distance to any other latent MTrP was selected in the upper trapezius muscle^17^ and then the practitioner therapist inserted the needle through the skin. Consecutively, the taut band was needled forward and backward until the exact punction point. Precise needling of the MTrPs elicited a brief contraction followed by relaxation of the muscle fibers; this is known as a LTR. Needling of the MTrP or the non-MTrP was repeated until 12 in and outs at a frequency of 1 Hz^2,17,23,24^.

### Statistical analysis

Data were analyzed using SPSS Statistics for Windows, version 25.0 (IBM, Armonk, NY, USA) was used for statistical analysis. A Kolmogorov–Smirnov test was used to test normality and no statistically differences were founded. A descriptive analysis was selected to summarize the outcomes in the 2 measurements in all groups (active MTrP DN group, latent MTrP DN group and non-MTrP DN group) and were included the mean, SD. A one-way ANOVA with the factor group (active MTrP DN, latent MTrP DN, and non-MTrP DN) was performed.

Repeated-measures analysis of variance (ANOVA) was performed considering the significance of the Greenhouse–Geisser correction when the Mauchly test rejected the sphericity to compare the differences between the 3 groups in the baseline. To analyze the contrast between the three treatment groups we used the ANOVA of repeated measures with 2 factors, 3 (groups) × 2 times (pre and post), only the pain intensity variable was analyzed in the interaction with the group factor the 11 times (pre, during, post, 1 h, 6 h, 12 h, 24 h, 48 h, 72 h, 1 week and 1 month) to include the calculation of the effect size, the partial eta (η^2^) coefficient was used.

Moreover, multivariate regression analyses were carried out using linear regression analyses by the stepwise selection method in order to predict the differences of the outcome measurements at different moments with respect to baseline which showed between-groups statistically significant differences in the prior described analyses. These outcome measurements (VAS, PPT trapezius, PPT tibialis Muscle, PPT expansion and Neck disability index) were considered as dependent variables. Independent variables were descriptive data, local twitch responses (LTR), patient’s pain recognition, group and the other outcome measurements at baseline. F probability was considered according to a P in = 0.05 and P out = 0.10.

All analyses were carried out “per intent-to-treat”. Moreover, a P-value lower than 0.05 was considered as statistically significant for a 95% confidence interval (CI).

#### Simple size

A total sample size of at least 18 subjects per group had to be recruited based on an a priori power analysis (G*Power 3.1.9.2 from the University of Düsseldorf). This power analysis was performed for the within-between interaction in a repeated measures analysis with 3 groups, 11 measurements (baseline, measurements during intervention, immediately after the intervention and measurements after 1, 6, 12, 24, 48, 72 h, 1 week and 1 month), a minimum power of 0.95, an effect size of 0.3 and an α level of 0.05. However, to cover possible losses, the sample size was increased by another 11 participants (n = 65).

### Institutional Review Board statement

The study was conducted according to the guidelines of the Declaration of Helsinki, and approved by the Ethics Committee of the University of Alcalá (CEID/HU/2018/41).

### Informed consent

Informed consent was obtained from all subjects involved in the study. Written informed consent has been obtained from the patient(s) to publish this paper.

## Results

### Clinical characteristics of the participants

**Figure 1:**
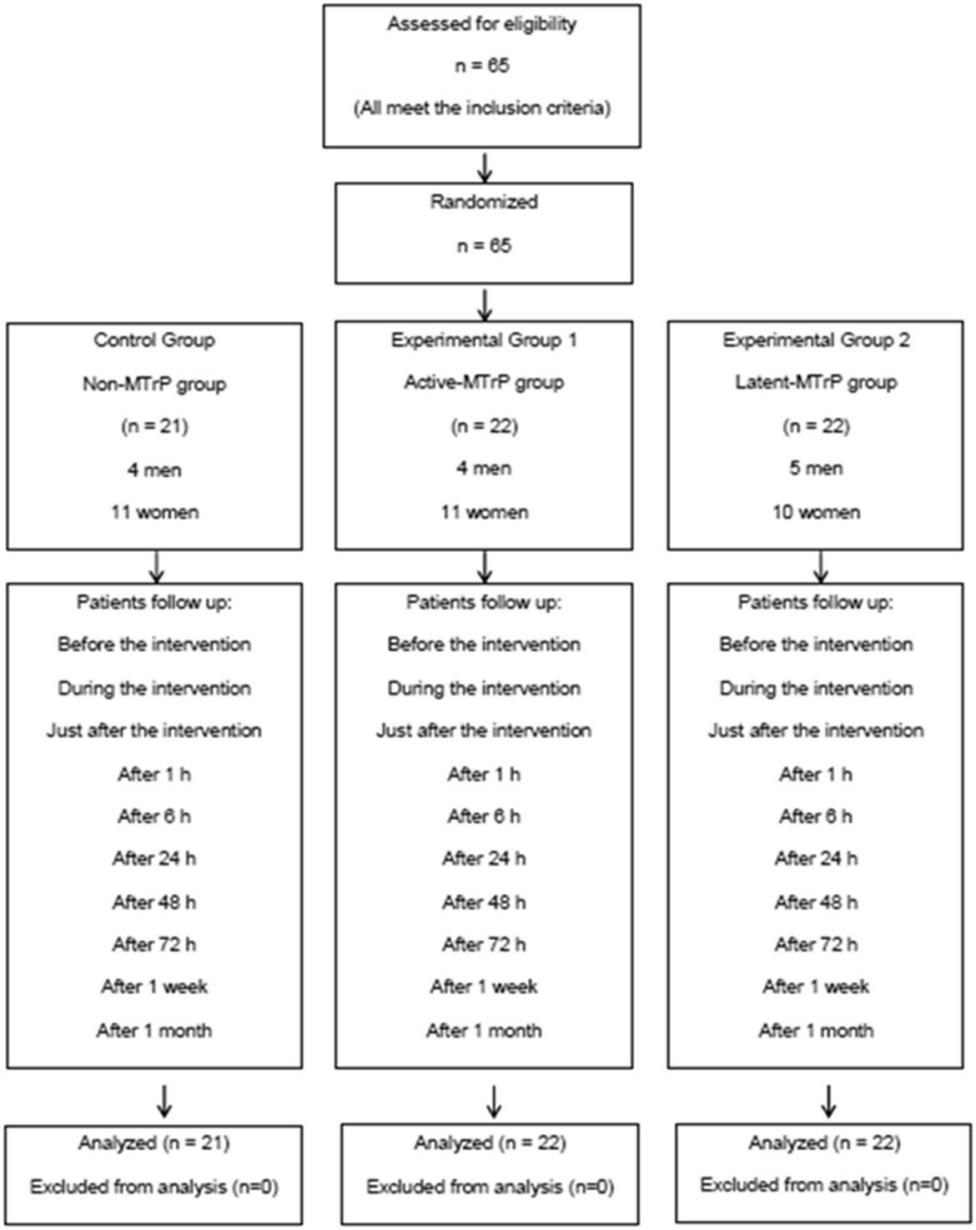
Flow diagram.

Sixty five participants were assessed for eligibility (Fig. 1). All subjects met the inclusion criteria. Therefore, sixty-five patients were included, of which 21 for the control group (Non-MTrP group), 22 for the Experimental group 1 (Dry needling in active-MTrP group), and 22 for the Experimental group 2 (Dry needling in latent-MTrP group).

There were not statistically significant differences between the 3 groups in terms of the demographic and clinical characteristics at baseline (Table 1).

**Table 1:** Demographic and clinical characteristics at baseline.

Demographics	Non-MTrP (n = 21)	Active-MTrP (n = 22)	Latent-MTrP (n = 22)	*P* value
Age (years)	27.90 ± 13.02	22.91 ± 7.14	28.45 ± 16.22	0.28
Sex (women/men)	12/9	12/10	11/11	0.89
BMI (kg/m^2^)	22.96 ± 3.34	22.94 ± 2.54	22.95 ± 2.31	1.00
Place (UAH/PHCC)	16/5	22/0	19/3	0.58
Lateralization (right/left)	16/5	18/4	17/5	0.89
IPAQ (low/medium/high)	2/10/9	0/10/12	0/11/11	0.98
VAS (0–10 cm)	6.19 ± 1.47	6.91 ± 1.44	6.73 ± 1.64	0.28
PPT in trapezius (kg/cm^2^)	2.84 ± 0.95	2.69 ± 0.76	2.90 ± 0.91	0.72
PPT in tibialis (kg/cm^2^)	4.92 ± 1.13	5.39 ± 1.36	6.06 ± 1.36	0.01*
NDI (0–50)	21.24 ± 13.56	20.68 ± 11.62	20.59 ± 13.01	0.98

Values are expressed as the mean ± standard deviation or number of participants.

*BMI* body mass index, *UAH* University Alcalá Henares, *PHCC* Primary Health Care Centre, *IPAQ* International Physical Activity Questionnaire, *VAS* Visual Analogue Scale, *PPT* Pressure Pain Threshold, *NDI* Neck Disability Index. *Indicates statistical significance P < 0.05.

### Pain intensity

**Figure 2:**
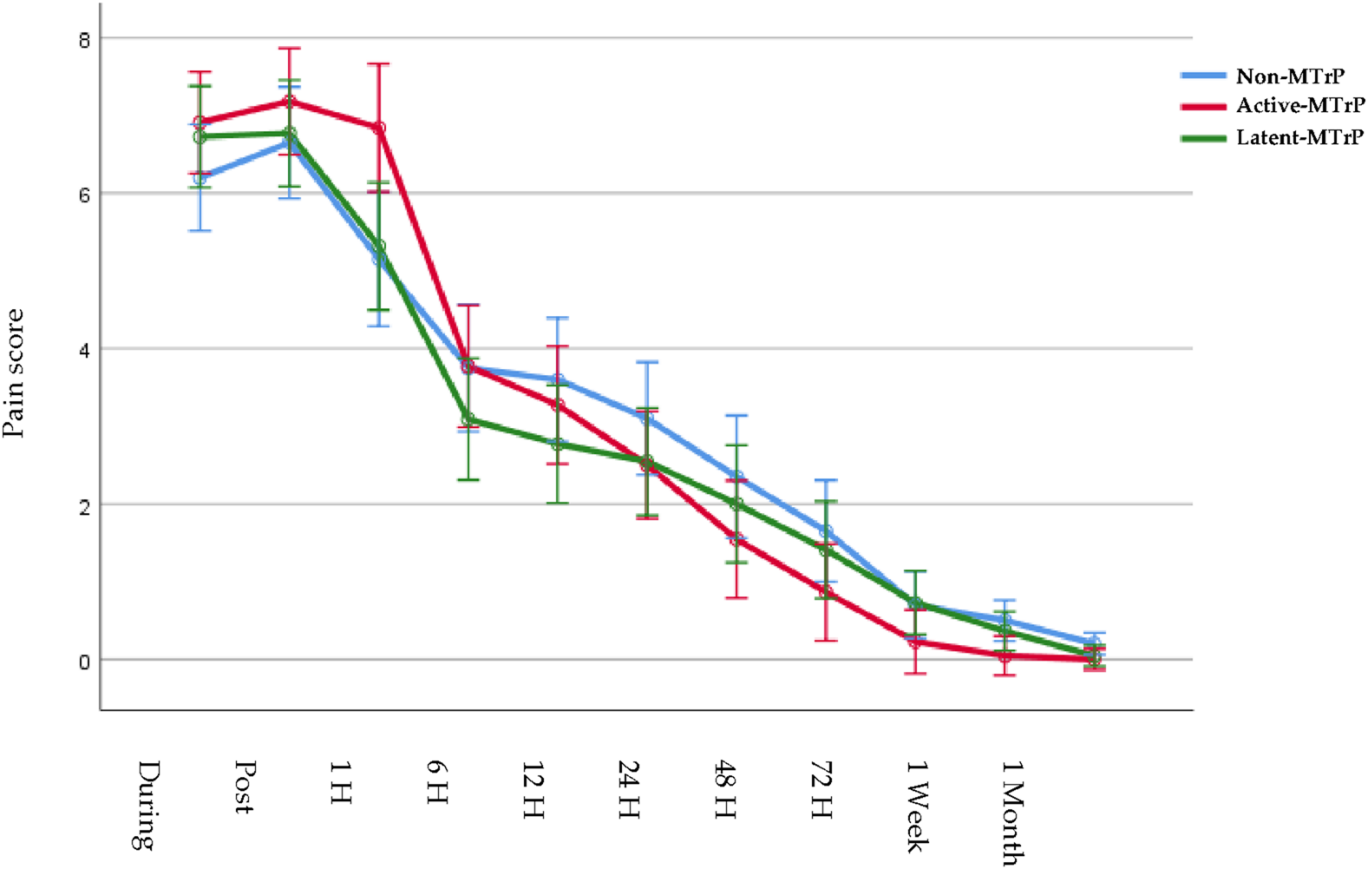
Pain intensity at 1 week.

The ANOVA showed a significant effect for time (F = 306.099; P < 0.0001; η_p_^2^ = 0.834) and demonstrated a significant interaction between group and time (F = 3.117; P = 0.0001; η_p_^2^ = 0.093) for changes in intensity of the neck pain, data presented in Table 2. Post hoc analysis showed that the DDN active MTrP group exhibited greater pain post intervention than non-MTrP group (outside to the MTrP) and DDN in latent MTrP group within the first hour post dry needling but not the rest of the times measured (P < 0.01). Furthermore, 1 week after needling, the DDN in active MTrP group showed lower pain scores than the non-MTrP group with DDN outside, see Fig. 2.

**Table 2:** Pain intensity outcome data and comparison between groups.

Variable	Non-MTrP	Active-MTrP	Latent-MTrP	Non-MTrP vs Active-MTrP	Non-MTrP vs Latent-MTrP	Active-MTrP vs Latent-MTrP
VAS PRE	6.19 ± 1.47	6.91 ± 1.44	6.73 ± 1.64	− 0.709 (− 1.875 to 0.457) 0.419	− 0.527 (− 1.693 to 0.639) 0.810	0.182 (− 0.956 to 1.320) 1.000
VAS during	6.65 ± 1.63	7.18 ± 1.65	6.77 ± 1.54	− 0.532 (− 1.755to 0.691) 0.866	− 0.123 (− 1.346 to 1.100) 1.000	0.409 (− 0.784 to 1.602) 1.000
VAS post	5.15 ± 1.46	6.84 ± 2.33*	5.32 ± 1.86	− 1.691 (− 3.158 to − 0.224) 0.019*	− 0.168 (− 1.635 to 1.299) 1.000	1.523 (0.091 to 2.955) 0.033*
VAS at 1 h	3.75 ± 2.07	3.77 ± 2.07	3.09 ± 1.27*	− 0.023 (− 1.419 to 1.373) 1.000	0.659 (− 0.737 to 2.055) 0.749	0.682 (− 0.680 to 2.044) 0.668
VAS at 6 h	3.60 ± 2.19	3.27 ± 1.78	2.77 ± 1.31	0.327 (− 1.026 to 1.680) 1.000	0.827 (− 0.526 to 2.180) 0.412	0.500 (− 0.820 to 1.820) 1.000
VAS at 12 h	3.10 ± 2.10	2.50 ± 1.26	2.55 ± 1.44	0.600 (− 0.635 to 1.835) 0.709	0.555 (− 0.680 to 1.789) 0.820	− 0.045 (− 1.250 to 1.160) 1.000
VAS at 24 h	2.35 ± 2.30	1.55 ± 1.41	2.00 ± 1.51	0.805 (− 0.538 to 2.147) 0.436	0.350 (− 0.992 to 1.692) 1.000	− 0.455 (− 1.692 to 0.992) 1.000
VAS at 48 h	1.65 ± 1.93	0.86 ± 1.13	1.41 ± 1.26	0.786 (− 0.326 to 1.899) 0.260	0.241 (− 0.871 to 1.353) 1.000	− 0.545 (− 1.631 to 0.540) 0.662
VAS at 72 h	0.70 ± 1.03	0.23 ± 0.53	0.73 ± 1.20	0.473 (− 0.259 to 1.204) 0.350	− 0.027 (− 0.759 to 0.704) 1.000	− 0.500 (− 1.214 to 0.214) 0.269
VAS at 1 week	0.50 ± 0.83	0.05 ± 0.21*	0.36 ± 0.58	0.455 (0.008 to 0.901) 0.045*	0.136 (− 0.310 to 0.583) 1.000	− 0.318 (− 0.754 to 0.118) 0.232
VAS at 1 month	0.20 ± 0.52	0.00 ± 0.00	0.05 ± 0.32	0.200 (− 0.042 to 0.442) 0.138	0.155 (− 0.087 to 0.396) 0.361	− 0.045 (− 0.281 to 0.190) 1.000

Values are expressed as the mean ± standard deviation. In comparison between groups: Difference of means, Confidence interval and P value according to Bonferroni’s correction. *Indicates statistical significance P < 0.05.

Statistically significant intra-group differences were found in the non-MTrP group and in the active trigger point dry needling group from one hour post dry needling to 1 month of follow-up and in the latent trigger point dry needling groups in the immediate post dry needling period (P < 0.01).

### Pressure pain threshold over trapezius muscle

**Figure 3:**
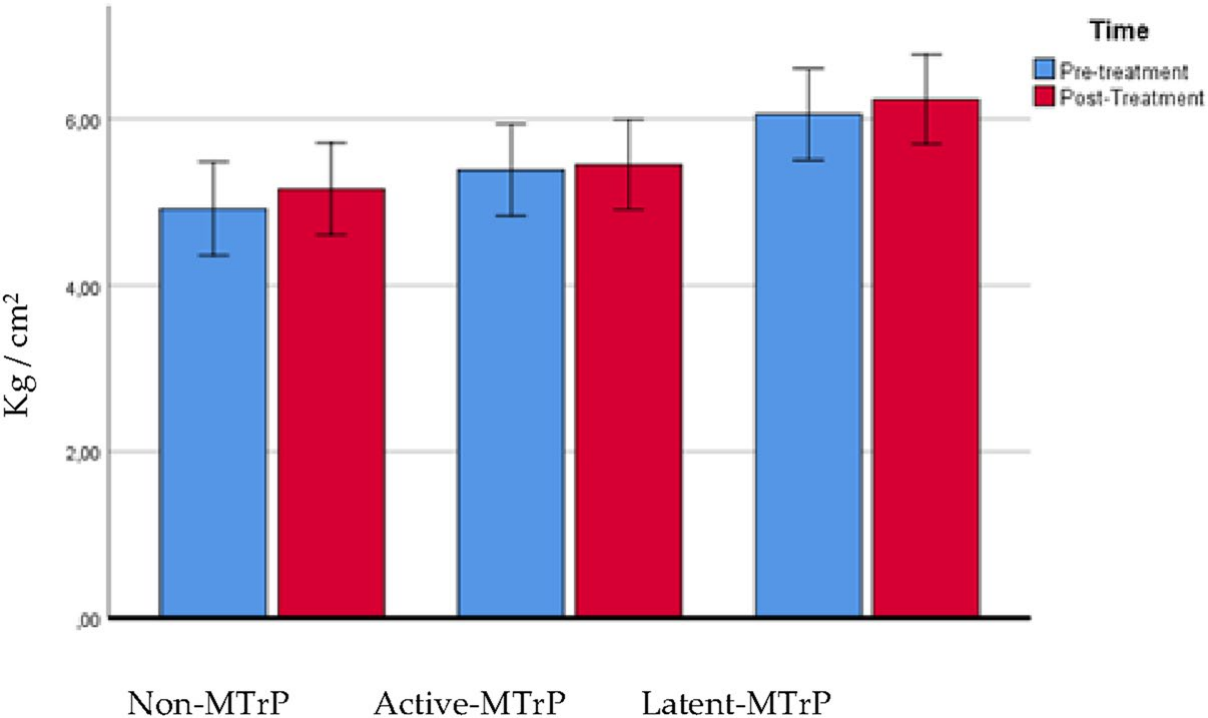
PPT trapezius muscle.

The ANOVA showed a significant effect for time (F = 57.607; P < 0.0001; η_p_^2^ = 0.482) but not statistically significant interaction between group and time were found (F = 0.157; P = 0.855; η_p_^2^ = 0.005) for changes in PPT in the neck, data presented in Table 3. See Fig. 3.

**Table 3:** PPT over trapezius muscle. Comparison between groups.

Variable	Non-MTrP	Active-MTrP	Latent-MTrP	Non-MTrP vs Active-MTrP	Non-MTrP vs Latent-MTrP	Active-MTrP vs Latent-MTrP
PPT pre	2.83 ± 0.95	2.69 ± 0.76	2.90 ± 0.91	0.144 (− 0.514 to 0.803) 1.000	− 0.067 (− 0.725 to 0.592) 1.000	− 0.211 (− 0.861 to 0.440) 1.000
PPT post	3.53 ± 0.98	3.44 ± 1.27	3.52 ± 1.45	0.096 (− 0.843 to 1.034) 1.000	0.008 (− 0.931 to 0.947) 1.000	− 0.088 (− 1.016 to 0.840) 1.000

Comparison between groups: Difference of means, Confidence interval and P value according to Bonferroni’s correction. *Indicates statistical significance P < 0.05.

### Pressure pain threshold over tibialis muscle

**Figure 4:**
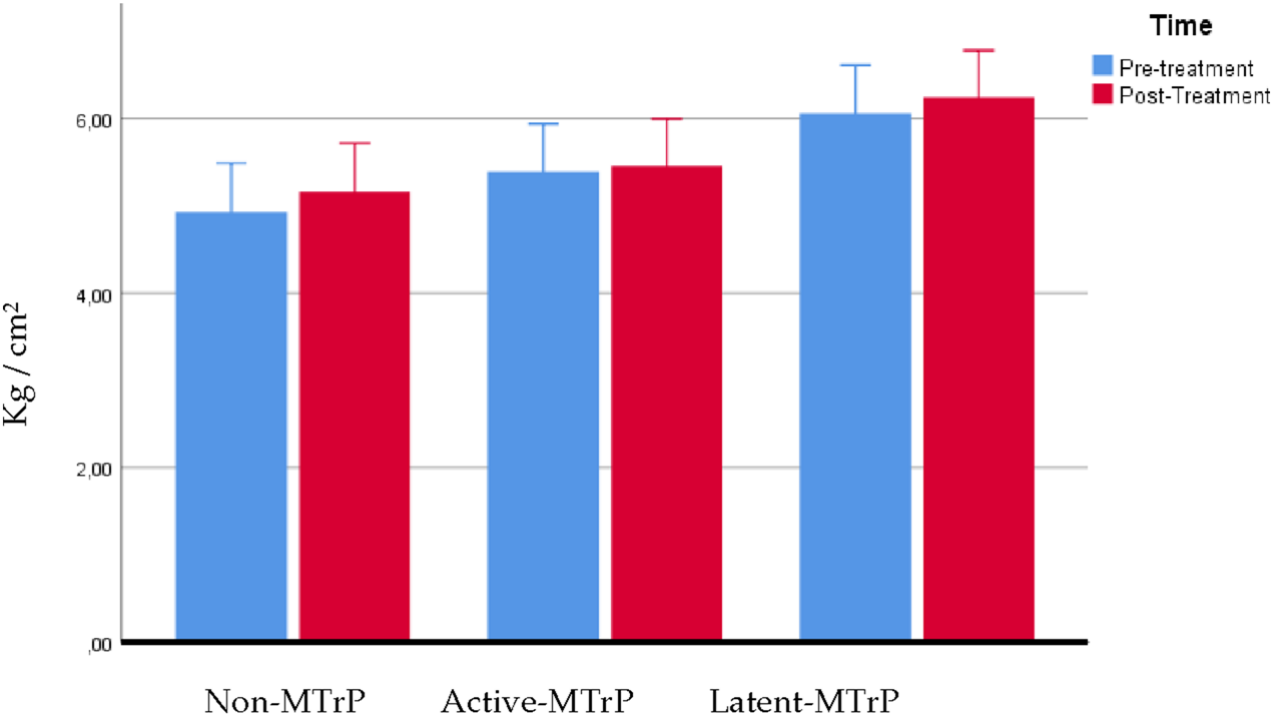
PPT over tibialis muscle.

The ANOVA showed a significant effect for time (F = 59,488; P < 0.0001; η_p_^2^ = 0.490) and demonstrated a significant interaction between group and time (F = 5.786; P = 0.005; η_p_^2^ = 0.157) for changes in PPT of the tibialis muscle, data presented in Table 4. Post hoc analysis showed that the DN active group exhibited lesser increase of pressure pain threshold post intervention than non-MTrP group (outside to the MTrP) and DN in latent group (P < 0.05), see Fig. 4.

**Table 4:** PPT over tibialis muscle. Comparison between groups.

Variable	Non-MTrP	Active-MTrP	Latent-MTrP	Non-MTrP vs Active-MTrP	Non-MTrP vs Latent-MTrP	Active-MTrP vs Latent-MTrP
PPT pre	4.92 ± 1.13	5.39 ± 1.36	6.06 ± 1.36	− 0.464 (− 1.435 to 0.506) 0.732	− 1.137 (− 2.107 to − 0.166) 0.016*	− 0.673 (− 1.632 to 0.286) 0.268
PPT post	5.16 ± 1.10	5.45 ± 1.34	6.23 ± 1.33	− 0.293 (− 1.241 to 0.656) 1.000	− 1.076 (− 2.025 to − 0.127) 0.021*	− 0.783 (− 1.721 to 0.154) 0.132

Comparison between groups: Difference of means, Confidence interval and P value according to Bonferroni’s correction. *Indicates statistical significance P < 0.05.

### Pressure pain threshold expansion

**Figure 5:**
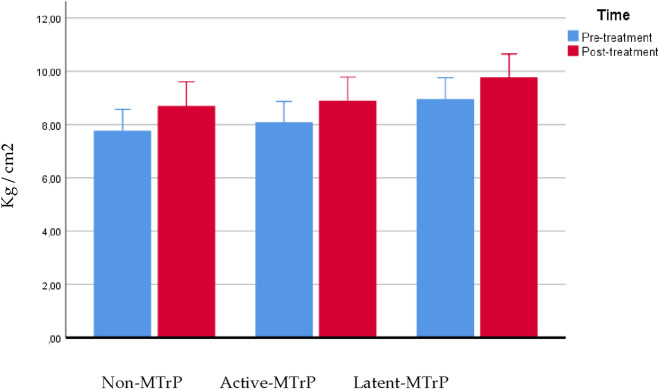
PPT expansion.

The ANOVA showed a significant effect for time (F = 79.842; P < 0.0001; η_p_^2^ = 0.563) but not statistically significant interaction between group and time were found (F = 0.202; P = 0.818; η_p_^2^ = 0.006) for changes in PPT summation expansion, data presented in Table 5. See Fig. 5.

**Table 5:** PPT expansion. Comparison between groups.

Variable	Non-MTrP	Active-MTrP	Latent-MTrP	Non-MTrP vs Active-MTrP	Non-MTrP vs Latent-MTrP	Active-MTrP vs Latent-MTrP
PPT pre	7.76 ± 1.87	8.08 ± 1.94	8.96 ± 1.73	− 0.320 (− 1.708 to 1.068) 1.000	− 1.203 (− 2.591 to 0.185) 0.111	− 0.883 (− 2.255 to 0.488) 0.354
PPT post	8.70 ± 1.90	8.89 ± 2.25	9.77 ± 2.08	− 0.197 (− 1.761 to 1.367) 1.000	− 1.068 (− 2.632 to 0.496) 0.294	− 0.871 (− 2.417 to 0.675) 0.511

Comparison between groups: Difference of means, Confidence interval and P value according to Bonferroni’s correction. *Indicates statistical significance P < 0.05.

### Neck Disability Index

**Figure 6:**
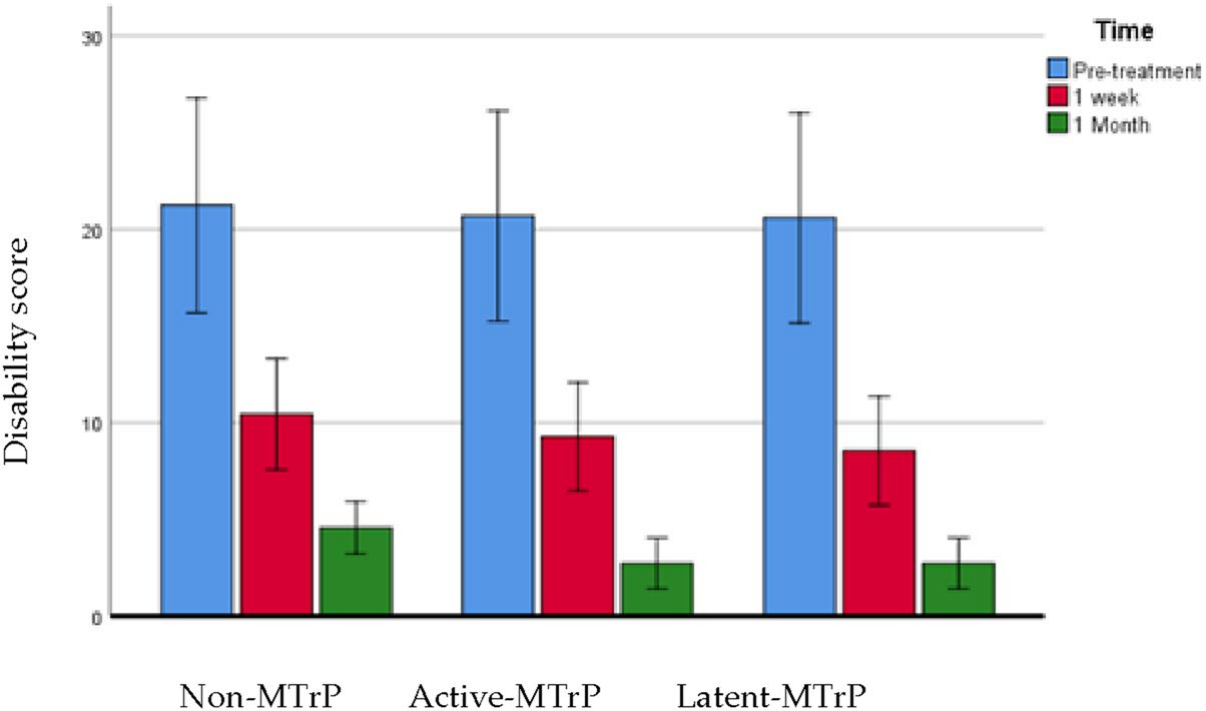
Neck Disability Index.

The ANOVA showed a significant effect for time (F = 131.775; P < 0.0001; η_p_^2^ = 0.680) but not statistically significant interaction between group and time were found (F = 0.098; P = 0.933; η_p_^2^ = 0.003) for changes in disability, data presented in Table 6. See Fig. 6.

**Table 6:** Neck Disability Index. Comparison between groups.

Variable	Non-MTrP	Active-MTrP	Latent-MTrP	Non-MTrP vs Active-MTrP	Non-MTrP vs Latent-MTrP	Active-MTrP vs Latent-MTrP
NDI pre	21.24 ± 13.56	20.68 ± 11.62	20.59 ± 13.01	0.556 (− 9.008 to 10.121) 1.000	0.647 (− 8.917 to 10.212) 1.000	0.091 (− 9.362 to 9.543) 1.000
NDI 1 week	10.43 ± 7.41	9.27 ± 5.22	8.55 ± 7.02	1.156 (− 3.801 to 6.113) 1.000	1.883 (− 3.074 to 6.840) 1.000	0.727 (− 4.171 to 5.626) 0.511
NDI 1 month	4.57 ± 3.96	2.73 ± 2.35	2.73 ± 2.80	1.844 (− 0.479 to 4.168) 0.166	1.844 (− 0.479 to 4.168) 0.166	− 9689E−16 (− 2.296 to 2.296) 1.000

Comparison between groups: Difference of means, Confidence interval and P value according to Bonferroni’s correction. *Indicates statistical significance P < 0.05.

### Association between the number of local twitch responses and pain provoked

There is no association that the higher the number of LTRs, the higher the percentage of subjects with moderate to severe neck pain (P > 0.05) but we found a statistically significant association that in the patients with the higher number of LTRs, it is also higher the percentage of subjects in whom their neck pain is reproduced (P < 0.05).

A greater association of a greater number of LTRs was found in the active and latent trigger point dry needling groups (P < 0.0001), than in the non-MTrP group.

### Association between the dry needling site and the provocation of your neck pain

In addition, we found an association of a higher percentage of subjects in which their neck pain is reproduced when dry needling was done at the active trigger point (77.3%) and in the latent (81.8%) (P < 0.001). However, no model was found in the prediction that producing their neck pain during dry needling produces a greater improvement in any of the variables measured.

### Multivariate regression analyses

#### Predictive model for the PPT in tibialis difference during DN versus baseline

PPT over tibialis muscle after DN with respect to baseline showed a statistically significant predicted model by bleeding after DN and place of dry needling (R^2^ = 0.218; β = − 2.356; F_[0.051]_ = 4.061; P < 0.05), indicating a prediction of higher mechanical hyperalgesia over tibialis muscle difference after versus before DN under the bleeding and site of dry needling (latent or active) difference. The rest of independent variables were excluded from the prediction model according to F probability (P in = 0.05, P out = 0.10).

#### Predictive model for the pain intensity difference at 1 week after DN versus baseline

Pain intensity difference at 1 week after DN with respect to baseline showed a statistically significant prediction model which was predicted by local twitch response (LTR) and side treated (R^2^ = 0.157; β = − 6.561; F_[1,63]_ = 4.410; P < 0.001), indicating a prediction of lower difference at 1 week after DN versus baseline under less LTRs and under side treated. The rest of independent variables were excluded from the prediction model according to F probability (P in = 0.05, P out = 0.10).

## Discussion

According to our study findings, pain intensity, disability and mechanical hyperalgesia were improved equally in all points that were treated with deep dry needling in the muscle, regardless of whether it was in a trigger point, latent or in an area where there is no trigger point. In terms of pain, it was seen that immediately post-treatment, the group that was received dry needling on the active trigger point, presented a worsening, but the group with the active trigger point was the one with the greatest reduction in pain at 1 week. In terms of mechanical hyperalgesia at a distance (tibialis muscle), the group with dry needling on the active trigger point had the least improvement.

Some previous works by Pecos Martín et al.^17^ have studied the importance of treating the specific site that produces pain. In the case of the present investigation, our objective was to achieve the maximum number of LTRs as it was found previously by the authors to be associated with a better clinical response. In those cases, better short-term results can be achieved for our patients as we have observed in our study. However, in contrast to the above, some studies indicate that performing DN without eliciting LTRs presented superiority over the DN along with eliciting LTR while the treatment aimed to receive long-term effects^7^.

Our results are consistent with another study in which dry needling was applied to the lumbar multifidus muscles in patients with low back pain. The presence of LTR did not produce a greater improvement in pain, disability, function and pain threshold to pressure at 1 week, although immediately the patients who had a LTR did have a greater improvement in function and pain threshold to pressure at 1 week^33^, showing that a local twitch response during dry needling is not always a synonym for successful treatment.

One of the initial studies comparing DN versus lidocaine infiltration found different results to ours, showing that mechanical hyperalgesia, pain intensity and range of motion improved more in the group that presented local twitch responses than the group that did not^14^. Although there are certain methodological limitations to this study, other researchers also suggest that the LTR produces a greater improvement^34^. Additionally, in the results obtained, we have been able to observe how this type of intervention produces not only an improvement in pain perception, but also in the pressure pain threshold and neck disability at short term. According to these findings, we found several articles that, making other approaches, but evaluating the same and other variables, also found improvements until the short term^17,35–38^. These findings are in part supported by the results of a study that found that pain relief correlated with LTR, but only when pain intensity was high^39^.

In relation with the pain intensity results, there is a greater soreness decrease after 1 week and 1 month, especially in the active MTrP group, as has been observed in previous studies, even over 2 points on the VAS scale when needling is done in the upper trapezius muscle^27,40–42^. This study showed a significant effect for time but not statistically significant interaction between group and time were founded for changes in PPT in the upper trapezius. On the other hand the DDN active group obtains a lesser improvement in PPT in the tibialis muscle, compared to performing DDN in latent trigger points or outside of trigger points in patients with neck pain. It has relation with other previous studies where researchers showed that PPT levels increase when DDN is done in an active MTrP^40^ and it could be a greater increase indeed after 2 days of the intervention^43^. The results obtained from mechanical hyperalgesia are in accordance with the conclusions of a recent systematic review which concluded that dry needling produces an increase in the pain threshold to pressure^44^. In a previous study by Llamas-Ramos et al. found that 2 sessions of dry needling produced a 73% increase in the pain threshold to pressure which was maintained for 2 weeks during follow-up^45^ and in a study of Pecos-Martin et al. the research found that dry needling on the active trigger point resulted in an increase in PPT of 53.5%, whereas dry needling outside the trigger point did not produce the same increase^17^. One limitation that we have found in our study in relation to the PPT in the tibialis anterior muscle is that in the group of DN in latent MTrP group the PPT was already higher than the rest of the groups and that is what may have influenced the statistically significant differences between the groups and therefore cannot be taken into account, although in reality what we have reported is the intra-group analysis, and in which we have seen that the group of DN in active MTrP group is the one that obtained the least change over time, regardless of whether that group had a higher or lower pain threshold in the tibialis anterior muscle. Pain intensity difference at 1 week after DN versus baseline was predicted by LTRs and side treated with a *R*^2^ coefficient of 0.157, indicating a significant prediction model but suggestion a low percentage of prediction. Lower pain intensity difference at 1 week after DN was predicted by lower production of LTRs during the DN procedure in accordance with a recent systematic review and meta-analysis suggesting a low level of evidence for immediate effects of obtaining LTRs during DN interventions on pain intensity^46^.

Cerezo Tellez et al. found a clinically relevant improvement of 4 points in PPT that was maintained for 6 months. In their research, they applied 3 sessions of dry needling in patients with neck pain. In our study we only obtained an improvement of 4.87%^47^. A possible explanation why other studies have found changes with a greater clinical effect may be due to the number of sessions or in the case of Pecos-Martin, because the dry needling was performed on the lower trapezius muscle despite only one session^17^. Lower PPT over tibialis anterior muscle after DN with respect to baseline was predicted by the presence of bleeding after DN and the place of DN with a *R*^2^ coefficient of 0.218, determining a significant prediction model although representing a low percentage of prediction. Bleeding was reported to be the most common minor adverse effect presented in the 16% of dry needling procedures^48^ but, to the authors’ knowledge, our study may be considered as the first trial claiming that the presence of bleeding may predict a lower PPT outside of the painful region after DN in patients with chronic neck pain.

The Neck Disability Index showed that all groups improved equally; therefore we can say that for the improvement of disability it does not matter where you perform the dry needling. The three groups have an improvement after 1 week and 1 month. This results are comparable to other studies where no differences were found between groups^24^.

So far, there are still studies showing that obtaining a LTR produces more immediate and lasting pain relief than not inducing LTR, as in a study where more patients were found with improvements in neck pain greater than those moderate clinically important differences observed when obtaining 6 LTRs and LTRs to exhaustion compared to not eliciting LTRs^23^. In our study, we found that there is a statistically significant association in relation to the greater the number of LTRs, the more frequently the symptoms of the patients are reproduced during the dry needling intervention (P < 0.05), although, on the other hand, we did not found an association that the more LTRs we achieved a greater clinical improvement. We have also obtained a greater number of LTRs when performing DDN on active and latent MTrPs, but that is not related to a major clinical improvement either. Other research also shown that the production of one or multiple LTRs during muscle MTrP DDN appeared to have a poor short-term correlation with pain and disability outcomes in patients’ neck pain^49^. The LTR during dry needling may be clinically relevant, but should not be considered necessary for successful treatment^33^. Relative to long-term interventions, DDN without causing LTRs has superiority over DDN with provocation of LTRs^7^. A greater association of a higher number of LTRs was found in the active and latent MTrP DN groups (P < 0.0001), than in the non-MTrPs group. The association was also found between the site where the dry needling was performed and the provocation of neck pain in the patients, finding a higher percentage of subjects in whom their neck pain was reproduced when the dry needling was applied to the active (77.3%) and latent (81.8%) (P < 0.001) MTrP. This finding is related to one of the recommended clinical criteria to identify active and latent MTrP, which is the appearance of LTR, in this case, during the intervention^8–10^. However, there was no model in predicting that producing your neck pain during dry needling would produce a greater improvement in any of the measured variables.

According to Hong^50,51^ the formation of the MTrP and the taut band (TB) is controlled by the central nervous system through a “MTrP circuit” and, thus, stimulation with a needle can transmit a strong signal to the central nervous system to induce the powerful reflex of the LTR that will help to reorganize the control that the central nervous system exercises over the MTrP and the tight band, breaking the vicious circle of the “MTrP circuit”. However, some recent study’s findings show that needling the MTrP of the soleus muscle specifically decreased stiffness measured at the MTrP but without any changes at an adjacent point within the same TB. However, when the TB was needled, no changes were observed in the TB or the MTrP. Such findings emphasize the importance of needling precision into the MTrP area to modify myofascial mechanical properties, although real effectiveness and effect are unknown in a clinical context^52^.

The rest of the mechanisms could explain the decrease in pain and the elevation of the pressure pain threshold that is usually observed in studies with DDN techniques as well as this current study. However, the known capacity of the autonomic nervous system to produce electromyographic changes in the MTrPs supposes of a possible action on the etiopathogenesis of MTrPs, which could justify motor effects, beyond the purely sensitive ones.

For all these reasons, it is essential to expand knowledge in basic research in order to continue making new findings in the experimental and clinical field that make us take not only a step, but also a leap in improving the treatment of our patients.

## Limitations

The current study has some limitations. We suggest that this study could be done on a larger sample size to provide more insight regarding the variables effects. The second is that we assess and treat the upper trapezius muscle in a single session, although other muscles may be involved in neck pain. Furthermore, the trapezius is one of the most intolerant to puncture and this prevents the results obtained from being extrapolated to dry needling in other muscles. Third, our study measured short-term effects; therefore, future studies should investigate the medium and long-term effects of deep dry needling by performing several treatment sessions on various muscles or by adding different locations of the same. The last possible limitation is the reliability of the identification of trigger points, as there is still a lot of controversy in the diagnosis and this may contribute to the fact that we do not find differences between the different groups of trigger points. Finally, PPT expansion was added in the present study in order to determine mechanosensitivity of both local and remote regions with respect the painful region, supposing that the summation may be a reliable measurement as the sum of PPT measurements of the upper trapezius and tibialis anterior muscles^31,32^. Nevertheless, future studies should consider reliable and useful measurements such as the temporal summation and conditioned pain modulation^53^.

## Clinical and research implications

The present study offers an idea of how to manage better the treatment of MTrPs using the DDN technique. Knowing a little more about the behavior of pain in relation to the place where we perform the technique, we can address the problems of our patients with more efficient treatments.

According to this study, patients have grater improvement if the physiotherapist applies the DDN technique in an active MTrP in relation with pain intensity but only for the first week, but not for a month, which means that in the short term it doesn't matter where you do the dry needling.

A greater contribution is necessary in this line of work in which the sample of participants is increased, as well as the number of sessions to be carried out, the muscles treated and a longer-term follow-up.

## Conclusion

Deep dry needling of the trapezius muscle, regardless of whether it is on a trigger point, latent or non-trigger point area, produces the same positive effects in improving pain intensity, discomfort and local mechanical hyperalgesia. In terms of mechanical hyperalgesia at a distance (tibialis muscle), the group with dry needling on the active trigger point had the least improvement. There is no association between the number of LTR and improvement and reproducing the patient’s pain during dry needling with the improvement of the patient.

It seems that precise location is not necessary when applying dry needling in clinical practice, but more studies need to be carried out with larger sample sizes, applying more sessions and with longer follow-ups.
